# Correction: Up-down biphasic volume response of human red blood cells to PIEZO1 activation during capillary transits

**DOI:** 10.1371/journal.pcbi.1008862

**Published:** 2021-03-31

**Authors:** The PLOS Computational Biology Staff

There is an error in reference 1. The correct reference is: Rogers S, Lew VL (2021) PIEZO1 and the mechanism of the long circulatory longevity of human red blood cells. PLoS Comput Biol 17(3): e1008496. https://doi.org/10.1371/journal.pcbi.1008496. The publisher apologizes for the error.
