# Supplementary material for: Up-down biphasic volume response of human red blood cells to PIEZO1 activation during capillary transits
Source: PLoS Comput Biol. 2021 Mar 3;17(3):e1008706. doi: 10.1371/journal.pcbi.1008706 (PMC7928492; doi:10.1371/journal.pcbi.1008706)

**APPENDIX**

**Figure A1. Accessing the red cell model (RCM).** The model programme and a comprehensive User Guide and Tutorial are available with full open access from a GitHub repository from the University of Glasgow (<https://github.com/sdrogers/redcellmodeljava>). This figure offers an introductory overview of the User Interface. **A: The Welcome Page.** The cartoon shows the main RBC components represented in model, including PIEZO1. At the bottom the user is offered two tags with alternative options to create a new simulated protocol, “New experiment” (on the left), or to transfer a previously saved protocol file “Load from file” (on the right). **B:** **The Central Page.** Contains two main panels and a set of six tags at the bottom. The left panel offers options for changing the initial (default) constitutive properties of the cell in the initial Reference State (RS). The right panel offers instructions for entering perturbations emulating experimental protocols and to implement the dynamic model responses. Protocol instructions are registered in sequential Dynamic State pages (DS). Bottom tags carry instructions for adding a DS stage, for running the model (Run), for saving a protocol file (Write protocol file), for seeking help with entries (Help), and for terminating the experiment (Close experiment). At the end of each run the user is offered options to plot any of the system variables for scrutiny or save the full output in a *.csv file.

**A**


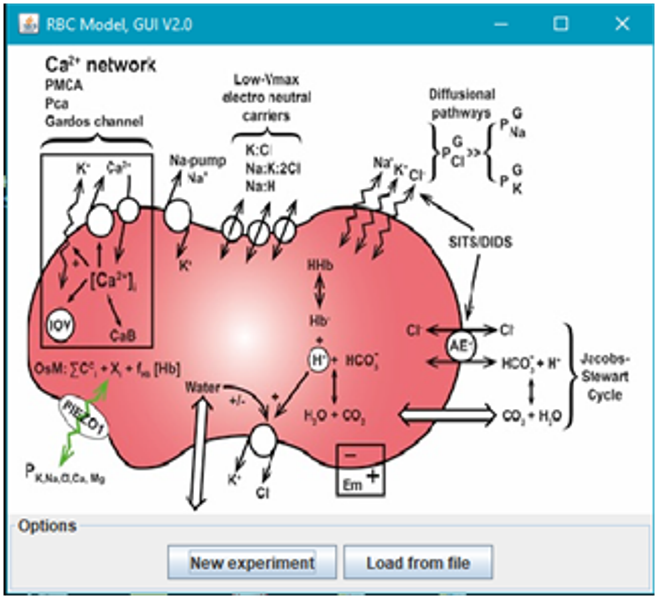


**B**


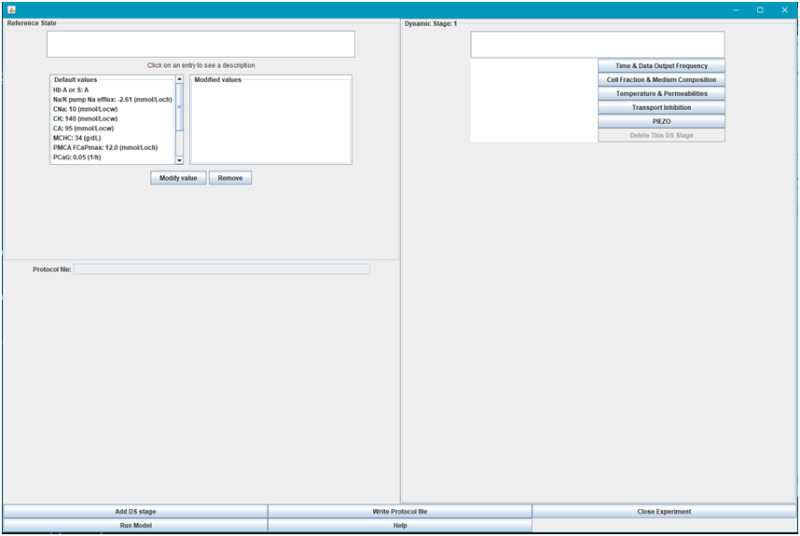

Supplement: S1 Fig — The model programme and a comprehensive User Guide and Tutorial are available with full open access from a GitHub repository from the University of Glasgow (https://github.com/sdrogers/redcellmodeljava). This figure offers an introductory overview of the User Interface. A: The Welcome Page. The cartoon shows the main RBC components represented in model, including PIEZO1. At the bottom the user is offered two tags with alternative options to create a new simulated protocol, “New experiment” (on the left), or to transfer a previously saved protocol file “Load from file” (on the right). B: The Central Page. Contains two main panels and a set of six tags at the bottom. The left panel offers options for changing the initial (default) constitutive properties of the cell in the initial Reference State (RS). The right panel offers instructions for entering perturbations emulating experimental protocols and to implement the dynamic model responses. Protocol instructions are registered in sequential Dynamic State pages (DS). Bottom tags carry instructions for adding a DS stage, for running the model (Run), for saving a protocol file (Write protocol file), for seeking help with entries (Help), and for terminating the experiment (Close experiment). At the end of each run the user is offered options to plot any of the system variables for scrutiny or save the full output in a *.csv file. (DOCX) [file pcbi.1008706.s001.docx]
