# Supplementary material for: Up-down biphasic volume response of human red blood cells to PIEZO1 activation during capillary transits
Source: PLoS Comput Biol. 2021 Mar 3;17(3):e1008706. doi: 10.1371/journal.pcbi.1008706 (PMC7928492; doi:10.1371/journal.pcbi.1008706)
Supplement: Attachment — Submitted filename: Response to Editor.pdf [file pcbi.1008706.s003.pdf]

Response to Editor, Dr Beard, on previous submission PCOMPBIOL-D-20-00715

We have reorganized and edited the material in the original papers following plan agreed with Dr Beard (PCOMPBIOL-D-20-00715), as follows:

1. **The original first paper was withdrawn. The new submission consists of twin papers, the first on the predicted homeostasis changes of human red blood cells during single capillary transits, the second on the changes during the 200000 or so capillary transits throughout the four month circulatory lifespan on the cells.**
2. **The material in the original first paper was comprehensively edited as a User Guide and Tutorial and deposited in an open GitHub repository together with the Red Cell and Lifespan models (RCM***.jar and Lifespan***.jar), all available with full access as explained in the new submitted papers. This provides reviewers and readers with easy access to the models for testing and replicating results reported in the papers, and for undertaking independent explorations and research.**
3. **The full set of governing equations of the core red blood cell model, together with explanatory text are now entered as an appendix to the first paper (Capillary transits).**
4. **The only experimental results in the literature approximating the dynamic in vivo events during capillary transits were used for a comparison between predicted and experimental outcomes, reported in figure 1 of the first paper.**
5. **Text sections in both papers were edited to make the topics more accessible and appealing to a general audience.**
