# Supplementary material for: Up-down biphasic volume response of human red blood cells to PIEZO1 activation during capillary transits
Source: PLoS Comput Biol. 2021 Mar 3;17(3):e1008706. doi: 10.1371/journal.pcbi.1008706 (PMC7928492; doi:10.1371/journal.pcbi.1008706)
Supplement: Attachment — Submitted filename: Reply to Reviewers-081020.pdf [file pcbi.1008706.s004.pdf]

Reply to Reviewer's comments

We are grateful for the positive and constructive comments from both Reviewers.

The replies are shown in numbered paragraphs following the original comments. The original Reviewers' comments are shown in Arial 10 bold, the replies in Arial 11 bold.

We indicate the papers addressed by the Reviewers comments in each reply as "First paper, Capillary transits", or "Second paper, Lifespan"

Reviewer #1.

The manuscript proposed a very interesting question about how red cell volume changed in response to Piezo1 activation during capillary transit. In this case, computational approach was used to simulate red cell volume upon piezo1 activation and demonstrated that cell volume increased following by shrinkage during capillary transit and the magnitude of such volume change was small.

Reply:
We appreciate the deft account of the question and the answer in the first paper!

Comments:

First paper, Capillary transits

1) The kinetic of such process is not clear. For example, start from time 0 where red cells enter capillary, how long it takes to activate piezo1 channel and what is the timescale in other related signaling pathways that eventually lead to cell volume change (figure 4)? This kinetic analysis is critical to understand the question proposed by the authors.

Reply:
As explained in the paragraphs headed "The reference protocol", "Ingress of a RBC in a capillary is simulated with an *instant* CVF transition from 0.00001 to 0.9 together with PIEZO1 activation.". The time scale of the pathways leading to volume changes is illustrated in Figs 3 and 6. These show almost instant onset to peak, peak durations of between a few ms to about ½ s, and durations of recovery phase varying greatly between a few ms to ~ 10s, all variations depending on the parameter values attributed to PIEZO1–mediated permeabilities, to PMCA Vmax, to JS-mediated cotransport rate, to Gardos channel PKmax or to combinations thereof. The mechanisms involved are discussed and analysed in detail in the text, and illustrated in the diagram of Fig 5.

On this kinetics issue the devil is in the details, and we see no way of bypassing the details in the analysis of Figs 3 and 6 and in the illustrated mechanisms of Fig 5 as explained in the current text. However we can improve the text of the paper to clarify the minimum level of precision the model had to operate in order to comply with the best experimental results available (Danielczok et al., [36]) to generate the correspondence shown in Fig 1. We edited the text of the first paragraph of Methods to stress the reversible nature of the processes involved, as follows:

"Their results strongly support a firm association between *reversible* RBC deformation, *reversible* PIEZO1 activation and *reversible* calcium influx during capillary transits, offering a unique opportunity to test the model capacity to emulate the calcium signal sequence by comparison with firm experimental results, as attempted in figure 1. It can be seen that the model can easily accommodate the pattern of the observed Ca^{2+} responses elicited by PIEZO1."

2) It is not clear to me how the magnitude of volume change was calculated. Doe it based on the number of piezo1 channels on the cell membrane?

Reply:
The magnitude of the volume change is entirely determined in the model by the magnitude of the permeabilities assigned to PIEZO1 at a fixed duration of the open state, as shown in Fig 3. The different permeabilities are meant to simulate the large range of variations observed in different experimental conditions. These conditions are also assumed to operate in vivo from differences in the extents of deformation RBCs experience while traversing capillaries affecting both the number of PIEZO1 channels activated and the duration of open-states. The important conclusion is that regardless of the PIEZO1 assigned permeability in the model, the up-down biphasic *pattern* of the volume response and its infinitesimal magnitude are constant features.

Second paper, Lifespan

In terms of the second manuscript entitled "PIEZO1 and the mechanism of the long circulatory longevity of human red blood cells", the description was comprehensive but it would be better if it could be more focused. For example, it would be interesting to focus on quantitatively how decay in different channel activities that regulated cell volume contribute to red cell longevity and what were the relative roles of each channel in this process.

Reply:
We assume the Reviewer meant "decay in the different *pump* activities". The quantitative effects of the parameters controlling the longevity pattern of RBCs are shown in Fig 5. Curves 1, 2 and 5 show the effects of varying the decay rate of the PMCA, and curves 1, 3 and 4 illustrate the effects of variations in the onset time of Na/K pump decay and on its rate of decay. These results show how relatively minor parameter differences have powerful quantitative effects. It is hard to decide between "comprehensive" and "focus" bypassing details. Still, we tried by editing the text in the Abstract to focus on the specific effects of pump decay, as follows:

"The first unexpected finding was that quantal density changes generated during single capillary transits cease accumulating after a few days and cannot account for the observed progressive densification of RBCs on their own, thus ruling out the quantal hypothesis. The second unexpected finding was that the documented patterns of RBC densification and late reversal could only be emulated by the implementation of a strict time-course of decay in the activities of the calcium and Na/K pumps, suggestive of a selective mechanism enabling the extended longevity of RBCs. The densification pattern over most of the circulatory lifespan was determined by calcium pump decay whereas late density reversal was shaped by the pattern of Na/K pump decay. A third finding was that both quantal changes and pump-decay regimes were necessary to account for the documented lifespan pattern, neither sufficient on their own. A fourth new finding…"

Reviewer 2. Dr Anna Bogdanova

Dr Bogdanova's comments address issues in the Guide and in both manuscripts.

1. I would like to thank the authors for taking time to develop a tool that is available for testing as an open-source software. I consider myself as a potential user of the software to try to design experiments of my own and try to predict their possible outcome. It is an interesting instrument that has to be tested. Experimental biophysics is in need of such modelling approaches if they prove valuable (it needs substantially more time to tell).

Reply:
We appreciate Dr Bogdanova's interest in testing the model and in recognizing the need of such tools in experimental biophysics. We also expect the new model applications to prove as valuable as the earlier ones did. The current applications were designed to explore the changes in RBC homeostasis during single capillary transits and throughout the circulatory lifespan of RBCs. The results essentially define our current knowledge of the circulatory behaviour of RBCs based on the body of early and recent information encoded in the model. Further refinements may be expected as additional information becomes available in the future.

2. However, some statements, such as a definition of initial condition as "a collection of RBC clones in plasma-like medium" make researchers involved in actual experiments worry already now. What we see suggests that the cells are no "clones" at all, and are not equipped with the same number of channels and pumps. As a result, we are dealing with a collection of sub-populations of cells with different properties, that include, but are not reduced to cell age. These sub-populations vary in abundance and maximal level of activity PIEZO1 channels, responsiveness to mechanical and chemical stressors and with different activity/abundance of PMCAs. We regularly observe "non-responders to stretch, making 10-40% of all cells. PMCA decay is not a continuous process as the enzyme, when undergoing cleavage by calpain, changes its activity in a "quantum" way, going from uncontrolled up-regulation in Vmax to its complete inactivation or loss, or incorporation into the inside-out vesicles (in cells of patients with sickle cell disease this process is very pronounces, as the author of this paper has successfully demonstrated a while ago). How does the model address this process, would it change a lot in the outcome?

Reply:
Thank you for pointing out the mention of "clones" in the Guide, a misleading description. We thought we had removed it from all texts, but obviously the "clones" in the Guide escaped our attention. We have now removed all mention of "clones" from the Guide.

The point Dr Bogdanova makes about RBC differences and about how our models deal with distributions and heterogeneities is important and applies to both papers. Let's consider first the case of a fresh RBC sample from a healthy adult. At the bench, experiments are performed on a cell population in suspension, with coefficients of variation (CVs) of between ~7% and ~13% in the relevant homeostatic variables (Blood, 1995, 86, 334-341). These CVs combine differences from endowments "at birth" and from cell aging. In the model, on the other hand, experiments are simulated on a RBC defined by the constitutive properties specified when entering the initial conditions. When using the mean values of a normally distributed cell population for defining the constitutive properties of the modelled cell, predictions and experimental results were repeatedly shown to agree to within a 5 to 10% margin in all measured variables, as expected from the observed CVs, extensively referenced in the Guide.

To study variability in more heterogeneous populations it is necessary to run multiple identical simulations on RBCs defined with the hypothesized deviations within the sets of parameters and variables considered in the model. Weighted proportions attributed to each variant within a defined distribution render predicted outcomes for the integrated response of the cell population under study. These can then be compared with experimental results to trace the nature of the hypothesized variant or distribution abnormality. This approach was demonstrated successfully with a metabolic model of RBC nucleotide metabolism (J. Gen. Physiol., 2011, 138(4), 381–391).

Dr Bogdanova stresses cell variations in PIEZO1 and PMCA as observed in different experimental conditions and asks "how does the model address this process, would it change a lot in the outcome?"

An important caveat before answering this question. In models of cellular homeostasis the performance of each cell component is represented by a kinetic equation. The nature of the molecular mechanisms causing variability only counts to the extent that its effects can be represented by kinetic parameters in the equations.

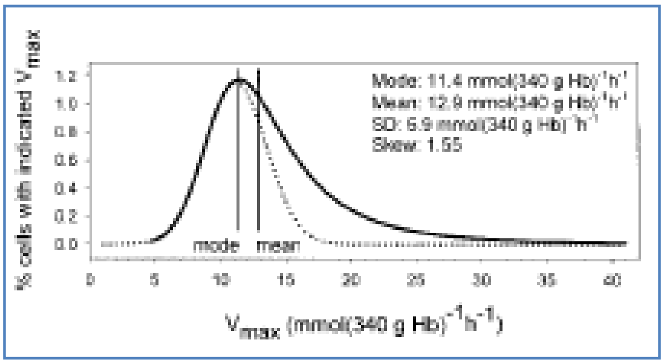

For instance, experiments showed that in RBCs from healthy adults PMCA Vmax varies as shown in the figure (Blood, 2003;102:4206-4213, Fig 4D). In Blood, 2007; 110:1334-1342, it was additionally shown that the huge Vmax variation was due to PMCA decline with cell age. For the results in the current modelling papers, this is the only information that counts for the kinetic representation of the PMCA. ***Whether the decline is due to glycation, cleavage by calpain or both is simply not addressed by the model. Discussing molecular mechanisms beyond model competence may convey the misleading impression that model results contribute information of value in that area*.**

The effects of PMCA and PIEZO1 variations on model outcomes was studied in detail as summed up below.

PMCA variations
Capillary transits (first paper). **The effects of PMCA variations within the observed Vmax range are shown in Fig 6. Their interpretation and complex mechanism is explained in the text and illustrated in Fig 5. PMCA Vmax variations within the measured range were shown to have powerful effects on the volume recovery phase of the biphasic response following PIEZO1 activation, and minor effects on initial peak height.**

***Lifespan model (second paper)*.** **As explained in the fourth paragraph under Results and Analysis, only an exponential decay function of the PMCA could deliver a right skew distribution, as measured (figure above). We have added a heading to the paragraph where this is reported in the paper to avoid confusion with the unrelated previous heading (Testing the quantal hypothesis). The new heading reads: "*Analysis of pump-decay patterns."* Unexpectedly, compliance with observed densification patterns could be implemented only with a very restricted set of pump-decay parameter values (Fig 4, curves 1, 7 and 8; Fig 5, curves 1, 5 and 6).**

PIEZO1 variations
Capillary transits (first paper). **The predicted effects of PIEZO1 variations on single capillary transits are shown in Fig 3; their mechanism is illustrated in Fig 4 and analyzed in detail in the text. The range of variations tested covered vast changes in PIEZO-mediated permeabilities (simulating combined number of channels x single channel conductance at fixed OS duration), with and without associated anion permeability changes. PIEZO1 activities strongly influenced the magnitude of individual cell responses, in line with Dr Bogdanova's comments and analysis,** ***but the predicted up-down biphasic pattern was always the same.*** **To stress this point, and to include updated information on RBC variability (Ref 55 now: Front. Physiol.**

11:392.doi: 10.3389/fphys.2020.00392), we added the following sentence in the first paragraph of the Discussion: "The most important conclusion from these results is that despite the large variations in the magnitude of the responses caused mainly by differences in PIEZO1 and PMCA activities [21, 55], the up-down biphasic volume pattern was always the same."

3. *Comment specific to material in the second paper, Lifespan*. When looking at the data in Fig 4,5,9 the changes in ion content occur slowly over time. I would expect them to occur for different cells at different time points and indeed (as shown in the examples of 1 and 4 in Fig 4) depend a lot of Ca2+ permeability for different cells. We have seen that free Ca2+ levels are not increase in the densest RBC population, but are very high in some cells (presumably those undergoing volume reversal) in the light RBC fraction. This is in contradiction to the inset of Fig 9 and with the notion that terminal swelling will increase the membrane surface tension opening the PIEZO1.

Reply:
The observations that free Ca2+ levels are very high in some cells apply strictly to RBCs in certain experimental conditions. There is no evidence at all that they apply to RBCs from healthy subjects in vivo. May be the pump-leak Ca^{2+} balance causes $[Ca^{2+}]_i$ to increase in aging RBCs but as far as we know there is no evidence that $[Ca^{2+}]_i$ is elevated beyond Gardos channel activation levels in vivo in circulating RBCs outside capillary transit periods. With our model we attempt to test the extent to which the body of reliable experimental evidence available can help account for in vivo dynamic events inaccessible to direct inspection or experimentation.

In this endeavour it is essential to apply a strict minimalistic approach in the representation of the RBC components in the model equations, with no free parameters, if possible. This implies avoiding inserting assumptions such as states of increased membrane surface tension activating PIEZO1 channels outside capilary transits periods, bound to "predict" known experimental outcomes without adding anything to their likelihood in vivo. There is no contradiction in the inset of Fig 9; the reason why the PIEZO1-induced peak $[Ca^{2+}]_i$ declines during density reversal is because of dilution as the cells in density reversal increase in volume while Ca^{2+} influx through PIEZO1 remains more or less the same during successive capillary transits.

4. "Can the model predict how many cells will be found at the terminal swelling stage in the whole population? Is there a "probability assessment" possible with it?"

Reply:
We are grateful to Dr Bogdanova for bringing up this important issue. It deserves further clarification in the paper. We thing addition of the following paragraph under "*The Reference Pattern"* heading in the fist paper answers Dr Bogdanova's question.

"The density distribution of normal human RBCs follows a Gaussian pattern with means around 1.085 – 1.095 g/mL [6]. Two conditions ought to be fulfilled in the model to comply with such a pattern. The first is that for most of the time the cells spend in the circulation their density should vary around the measured population mean. This implies a dominant period of gradual densification of "middle age" cells (~ between days 20 and 100 of circulatory age in the model, Fig 3), with younger and older cells in lower and higher density states, respectively, contributing lesser proportions of aging time. The second condition is that for the Gaussian pattern not to be significantly altered at the high density end, the contribution of the oldest cells undergoing density reversal must be limited both in their proportion and in the extent

of density reversal, a powerful constraint on the model parameters controlling Na/K decline, onset time and rate of decline."

5. How is a membrane loss kinetics component addressed in the model?

Reply:
This issue was not addressed at this stage. The current model assumes constant membrane area throughout RBC lifespan. The magnitude and pattern of age-related membrane area loss is poorly characterized in normal RBCs. It may be of substantial relevance for sickle cells in hyperdense collapse. This issue deserves further investigation before modelling may be expected to contribute useful new insights.

6. How is the amount of bound water (mainly Hb-bound) that cannot participate in diffusion or volume regulation integrated into the model? This is a substantial fraction that changes with the changes in RBC volume.

Reply:
While working on the first version of the red blood cell (RBC) model (J. Membrane Biol. 1986, 92, 57-74) back in the early eighties, we tried to assess the possible role of bound water. We consulted Max Perutz who sent a preprint, now available for downloading from BioSistems (Perutz, M.F., BioSystems, 8 (1977) 261-263. The role of bound water in haemoglobin and myoglobin).

Perutz's results point to ~90 bound water molecules per haemoglobin tetramer. With this figure it was easy to calculate the approximate fraction of bound water in human RBCs. There are about ~$5*10^{-3}$ mol of Hb tetramers per litre cells. With 90 water molecules bound per tetramer, we have $90*5*10^{-3}$ = 0.45 mol of bound water molecules per litre cells. The approximate volume of cell water per litre cells is ~0.75 litre, equivalent to 42 mol of water per litre cells. The fraction of bound water is therefore 0.45/42 ≈ 0.01, a value much smaller than that originally suggested by Adair & Adair, and too small for meaningful influence on the osmotic driven fluid flows considered in the RBC model. The scope of the bound water effects contrast with the powerful effects of haemoglobin crowding on cell dehydration, effects accurately predicted by the model. This is relevant to the bound water issue, because crowding acts to prevent levels of RBC dehydration which could otherwise cause bound water to reach a 5% level. The crowding effect and its mechanics is explained, demonstrated and referenced in detail in the User Guide.

Bound water is obviously extremely important for proper protein function at a molecular level, but quite irrelevant at the phenomenological level homeostasis models operate. The excellent quantitative agreement between predicted and measured cell volume, pH, membrane potential and ion content changes found by us and others in a large variety of experimental conditions confirms a level of bound water influence below detection within the ~5-10% margin of agreement between model predictions and experimental results documented in the past.
