# Supplementary material for: Up-down biphasic volume response of human red blood cells to PIEZO1 activation during capillary transits
Source: PLoS Comput Biol. 2021 Mar 3;17(3):e1008706. doi: 10.1371/journal.pcbi.1008706 (PMC7928492; doi:10.1371/journal.pcbi.1008706)
Supplement: Attachment — Submitted filename: Reviewer3 replies 261120.docx [file pcbi.1008706.s005.docx]

**Simon Rogers and Virgilio Lew**

**25 November 2020**

**MSs: 20-01635_R1 & 20-01636_R1**

**Reply to Reviewer#3.**

(1) Does their java code correctly implement the model equations?

**REPLY: The code was thoroughly tested throughout development side by side with the original PowerBasic version and gave identical results to machine precision. These tests were performed over a series of protocols that were designed to expose all parts of the code confirming the full implementation of the model equations.**

**(2)** Are their model equations accurate descriptions of the biological processes they intend to represent?

**REPLY: The pre-PIEZO1 model was a minimalistic representation of RBC homeostasis with the demonstrated ability to predict RBC behaviours in a vast range of physiological, pathological and experimental conditions to a semi-quantitative level. These are the best credentials for the model equations to provide accurate descriptions of the biological processes they intend to represent.**

**In the User Guide we provide a sample of the most counterintuitive and unexpected model predictions, fully validated in different laboratories. These have helped solve issues of fundamental relevance on volume, osmolarity, ion contents, ion fluxes, membrane potentials and pH control in normal and sickle RBCs and also in malaria-infected RBCs. The PIEZO1-extension builds on this solid background using a parameter set and kinetics firmly based on selected experimental data gathered over the last decade, as explained in the manuscripts. Reviewer#2 peer-reviewed the equations of the PIEZO1-extended model.**

**Question (2) suggests that Reviewer#3 is unaware of this body of research, central to the RBC homeostasis field and to the research under review.**

**(3)** Do those equations collectively describe enough biological processes to enable accurate prediction of in vivo changes in RBC volume during circulation?

**REPLY: The predictive power demonstrated by the basic model in its multiple previous applications, all experimentally verified, never falsified yet, sets a strong background for expectations that the predictions from the current PIEZO1 extension have a reasonably high probability of success in delivering a realistic picture of the circulatory homeostasis of hRBCs.**

***Methodologies for testing these predictions do not exist yet.* Only future experimental tests could provide final answers. Many of our previous model predictions experienced variable delays for their final experimental validations. The original 1986 predictions of cell-acidifications associated with K^+^ permeabilizations were indirectly confirmed in 1987, but it took 24 years (*J Physiol* 588.24 (2010) pp 4995–5014) for an Oxford lab to come up with the new indicator methodologies that fully validated these and other predictions of the original model. The most recent case concerns the volume changes predicted for the malaria parasite Plasmodium falciparum and for its host RBC throughout the full 48h reproduction cycle of the parasite. Our 2003 model predictions (Blood, 2003; 101,4189-4194) were fully confirmed 14 years latter in a Heidelberg lab by methods we could never have anticipated would be developed to the required level of precision at the time (Cellular Microbiology 2017; 19: e12650; DOI 10.1111/cmi.12650).**

**On “*enough* biological processes”, we need to explain the minimalistic approach we apply in our model building. The initial model structure was built around the minimal set of component processes with the most solid body of experimentally supported data needed to explain all known behaviours at that time. Candidate new components or extensions were considered for incorporation only if the previous structure failed to account for new experimental results, regardless of the strength of literature supporting the new candidates.**

**In the Guide we provide an example of evidence for the existence of a Ca^2+^-activated electroneutral K^+^:H^+^ exchanger in the plasma membrane of RBCs from different species and also in the plasma membrane of certain tumour cell types, very actively researched in different labs in the 70s and 80s. Our original 1986 basic model, without this transporter, nicely recapitulated the experimental observations used to support the presence of this transporter in human RBCs, a result that surprised us all because no one had anticipated the mechanism revealed by the basic model to explain those observations (Guide and Refs 64-66). After a decade of fading controversies, the Ca^2+^-activated electroneutral K^+^:H^+^ exchanger finally disappeared from the literature. Had we placed it in the model we might still be chasing a ghost.**

**Back to “enough biological processes”. So far, the answer is yes.**

For (3) readers need volume measurements to validate simulation results. None are provided. The poor fit to experimental data in figure 1 is explained in the authors’ response as something other than experimental validation of their model. It seems that there is no attempt to validate their model of the effect of shear stress on RBC volume with experimental data. Given many modern methods for measuring RBC volume, this model and its surprising claim must be validated.

**REPLY: We fully agree with the desirability of experimental tests of model predictions. However, as explained above, we are not aware of any “modern methods” that could measure RBC volume changes during capillary transits in vivo. This is why “none are provided”. When “modern methods” develop to the required optical resolution, and permit the non-toxic use in vivo of indicators to record the dynamic variations in volume or associated calcium or pH signals needed to test model predictions, then perhaps the retinal microcirculation may become a viable testing ground in human volunteers.**

**Lacking any specific suggestions, the Reviewer’s comments imply that experimental tests are current feasible seeding the false impression that we failed to validate our surprising claims by neglecting such tests. “None are provided” is the proper qualifier for the Reviewer’s unspecified “modern methods” remark.**

As noted in the first review, the authors make many simplifying assumptions and limit their focus to a narrow range of conditions, for instance only considering events that occur in capillaries, ignoring mechanical effects, cellular material properties, and different conditions present in vivo, for instance, in arterioles where shear rates are much higher, or pulsatile flow. They write in their reply that a justification for the narrow focus is provided in the “Preliminary RCM tests” section, but there is no mention of mechanical forces, other vessels, or pulsatility. It is an enormous stretch to ask readers to believe that a model devoid of mechanics can accurately predict a volume change on the order of 10e-5.

**REPLY: The realms of circulatory mechanics the Reviewer mentions concern rheology, not RBC homeostasis. PIEZO1 is the only known transducer of mechanical effects into ion traffic relevant to RBC homeostasis. The Reviewer keeps confusing rheology with homeostasis ignoring previous polite explanations.**

**The prediction of a volume change on the order of 10e-5 was a big surprise to us as well, the first of the four major unexpected surprising findings in our papers. The 10e-5 prediction came out of the model-encoded representation of the true and tested processes that control RBC homeostasis. It knocked out many long-held misconceptions, by most of us in the field, the quantal hypothesis among others. But based on current knowledge we cannot fault the mechanism behind these surprises, as revealed by the model and explained in detail in our papers.**

The first review pointed out several misleading oversimplifications in the “primer” section, including overly narrow claims of surface-to-volume ratio, claims about gradual densification that contradict references cited, and skewed summary of mechanisms of RBC turnover. They revise their statement on RBC turnover but still only mention one mechanism. For the other oversimplifications, the authors reply that these details are not needed in a primer, but the primer is part of a scientific paper where accuracy and balanced reports of current knowledge are essential.

**REPLY: We stand by the correctness of our brief statements and for the length-value balance assigned to each within the primer. On the three specific examples provided: we could not find any “claims of surface-to-volume ratio” anywhere in the text of the papers or Guide. Which “contradictions” with Refs 8-13 is the Reviewer referring to? We couldn’t find any. Gradual densification of hRBCs for most of their lifespan is the most solidly established and universally accepted fact in the hRBC literature. Are these the “misleading oversimplifications”?**

**With regard to “skew”, the Reviewer appears to be pressing for a different skew he/she favours. We agree that it is important to mention the existence of a terminal clock in the primer but we see no reason to enter into controversies over its mechanism when this issue is not addressed in the papers. *We have reduced the text but retained the references, including those suggested by the Reviewer, for the benefit of readers.***

The authors were asked to justify their statement that Danielczok et al. reports “the closest experimental approximation to capillary flow conditions available to date.” They explain that Danielczok studies PIEZO1 in a unique way, but their revised manuscript does not clarify this point and still justifies the sole focus on Danielczok based on “capillary flow conditions.” They also explain that they are not using the Danielczok data for validation, which leaves no experimental validation.

**REPLY: Our amended text explained that Danielczok’s** **results strongly support a firm association between reversible RBC deformation, reversible PIEZO1 activation and reversible calcium influx during pressure-driven flow through a capillary-size constriction in a microfluidic channel. These are the essential intermediate stages in PIEZO1 mediation of the critical calcium effects during capillary transits that we chose to represent in the model. This fully clarifies the reasons for this choice. As for the “sole focus”, we would be very grateful if the Reviewer could find any *other* result in the literature that approximates this level of experimental information relevant to the PIEZO1-calcium interaction during capillary flow.**

**Fig 1 simply demonstrates that the PIEZO1 phenomenology built into the model can emulate observed experimental behaviour using protocols with time-courses and external calcium concentrations substantially different from those investigated for the in vivo conditions. If the model had not been able to emulate a [Ca^2+^]_i_ increase period for the specific experimental conditions of the Danielczok experiments, *this would have falsified our model, a critical test of model viability, not of the model predictions concerning capillary flow.***

**As explained above, experimental validation of the in vivo condition is not currently feasible. The comment that “they are not using the Danielczok data for validation, which leaves no experimental validation.” *misleadingly insinuates that a currently feasible experimental validation was not implemented.***
